# Evaluation of medical consultation letters at King Fahd Hospital, Al Hufuf, Saudi Arabia

**Published:** 2012-06-29

**Authors:** Hamed Abd Allah Al Wadaani, Magdy Hassan Balaha

**Affiliations:** 1 College of Medicine, King Faisal University, Saudi Arabia

**Keywords:** Medical consultation, referring patients, Saudi Arabia

## Abstract

**Background:**

In surgical wards, it is of paramount importance to communicate with other health care providers, mostly physicians, referring patients to them for their consultation on any health conditions that affect pre-operative, operative and post-operative patient care. The purposes of this investigation were to assess the appropriateness of physician responses in medical consultation reports and compare physician responses when using these reports from different levels of health care providers.

**Methods:**

This study was conducted in Al-Hufuf, Saudi Arabia. The researchers evaluated all the surgical consultation letters in the files during the period between March 2010 and March 2011. From the explored 234 files, only 200 consultation letters were chosen as there was a referral data plus consultation data in the same file. We evaluated the quality of consultation report included the ethical concerns towards colleagues and patient, consideration of patient safety in all opinions, comprehensive pertinent scientific information, addressing the patient's medical condition with putting possible differential diagnosis, conclusion and precise management plans suggested.

**Results:**

The results showed that the specialists' consultation letters had the highest percentage of fulfillment of all the six items in the consultation report. There is no uniform existing consultation report form.

**Conclusion:**

Specialist form showed the highest number of mentioning the diagnosis. Consultant form showed the highest number of mentioning the concise aim of referral. The highest percentage of all categories mentioned all items in consultation report with a good level were the specialists.

## Background

Surgical patient population becomes more medically complex and the current health care providers become more aware about the need for syndromic diagnosis and multisystem disorders. It is of paramount importance to communicate with other health care providers, mostly physicians, referring patients to them for their consultation on any health conditions that affect pre-operative, operative and post-operative patient care. However, both older and more recent studies indicate that opportunities for good communication are commonly missed [1]. Poor communication may result in delayed diagnosis, inadequate follow-up, erosion of patient confidence and increased costs through duplication of services [2].

Unfortunately, some consultation requests simply ask the physician for either medical clearance, i.e. “any contraindications for some surgical procedures” or guidance in preoperative preparation. Patient care may be delayed needlessly as pertinent information had to be obtained by resubmitting consultation requests or contacting physicians by telephone. Unnecessary consultation requests and nonspecific referral might lead medical consultation requests to be inadequate. This might be due to lack of knowledge on the needed relevant information and improper request writing [3, 4].

The 2005 CanMEDS Physician Competency Framework with its 7 Roles and 28 key competencies forms the basis for the development of specialty specific objectives of training, which each and every discipline that is recognized by the College is required to develop. This framework represented the basis of some of the recent medical curricula. In Saudi Arabia, the Saudi Meds framework was proposed in 2010 by multiple health professionals in the Kingdom. It provides a competence-based framework that reflects the principles of professional medical practice in Saudi Arabia. This includes the general competencies expected of medical graduates and the essential learning outcomes for undergraduate medical education. Saudi Meds is proposed as a national framework that promises equivalent standards, while at the same time guaranteeing schools and faculty's autonomy. In both frameworks, communication skills represented one of the main competencies [5, 6].

All the proper communication in the health sector is done through the good role model and guidelines of health care system in Saudi Arabia. There is no current published system of training or reminding on the value of this important communication parameter in the medical field. To the knowledge of the authors, there are no published studies regarding neither medical referral nor medical consultation in Saudi Arabia.

We hypothesized that the CR would contain uniform valuable information which is beneficial to the patient condition. Also, we hypothesized that the CR would allow a streamlined process of obtaining the appropriate information with more senior health care providers. The purposes of this investigation were to assess the appropriateness of physician responses in medical consultation reports and compare physician responses when using these reports from different levels of health care providers.

## Methods

This study was conducted in Al-Hufuf, Saudi Arabia. Al- Hufuf is the Capital city in Al-Ahsaa; the largest province in the Eastern region of Saudi Arabia. It is home to more than 1.5 million people. Al Hofuf King Fahd Hospital (HKFH) represents the central pooling hospital in Al-Ahsa Province. This hospital is a referral center providing secondary and tertiary level of care serving the population of Al Ahsa.

The data used in the current study were derived from the HKFH files after permission from the hospital authorities while maintaining file and patient confidentiality. Study protocol as well as the data collection forms was approved by the ethics committees of our institution and the HKFH. The design of this research was a cross sectional study. The researchers evaluated all the surgical consultation letters in the files during the period between March 2009 and March 2011.

**Figure 1:**
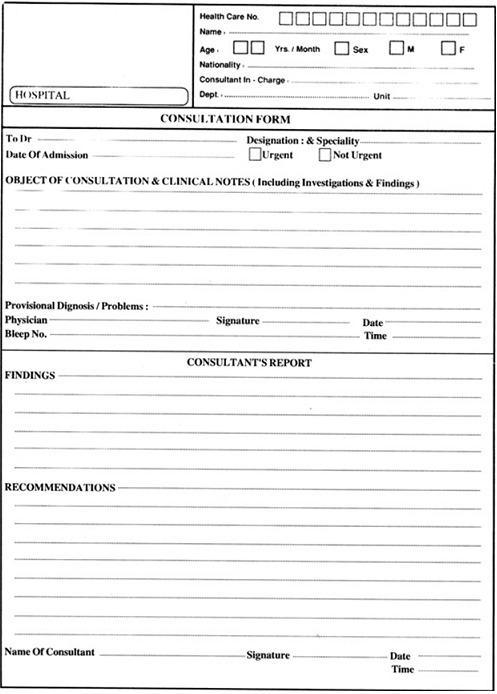
Consultation form used in King Fahd Hospital and studied in this research

The hospital used a structured consultation form (Figure 1) which contains a lot of referral information and an area for consultation. The upper right area of the form contains the patient information including name, age, nationality, responsible physician, hospital number and her inpatient place and number. The middle part contains referral data including referring person, speciality, type of referral, provisional diagnosis and aims of referral. The lower part is for consultation and is divided into findings and recommendation sections.

The chief nurse was asked to prepare a chart of the patients for whom surgical consultation requests had been sent to physicians. The patient records were retrieved, and the copies of the consultation letters in the records were examined after covering of the patient name. All the files were completely explored and checked by the interns working in the hospital under first author supervision. A semi structured form devised by the authors was used to record the data; which include patient pertinent data, referring physician, clinical referral information and data about quality of consultation letter. From the explored 234 files, only 200 consultation letters were chosen as there was a referral data plus consultation data in the same file.

We evaluated the responses of the physicians for appropriateness through consensus. Criteria for this evaluation, was based on referral document assessment and consultation report assessment. Source of consultation in our study was classified according to physician seniority into consultants, specialists and residents.

Referral document was assessed for inclusion of basic patient information, details of referring physician, description of clinical condition and the need for consultation. Quality of consultation report included the ethical concerns towards colleagues and patient, consideration of patient safety in all opinions, comprehensive pertinent scientific information, addressing the patient's medical condition with putting possible differential diagnosis, conclusion and precise management plans suggested. Data Analysis

The information in CRs was tabulated and descriptive statistics were generated for each item for each of the consultation report writers. Comparison was done based on the seniority level of consultation. Chi-square analysis was used to evaluate the significance of difference in numbers of appropriate obtained responses.

## Results

Basic pertinent patient information was presented in Table 1. They include name, age, sex, medical record number and location of the patient in the hospital. Details related to the referring physician included his name, signature; date and speciality were presented in Table 2 as classified by the seniority level of the referring physician. Non uniform referral reports were presented.

**Table 1:** Details of referring Physician

Source of consultation	Consultant N = 40	Specialist N = 78	Resident N = 82
**Item**	**No (%)**	**No (%)**	**No (%)**
Specialty	31 (77.5)	49 (62.8)	50 (61.7)
Date/ time	33 (82.5)	68 (87.2)	65 (80.2)
Name	38 (95)	65 (83.3)	57 (70.4)
Signature	40 (100)	74 (94.9)	68 (83.9)

**Table 2:** Analysis of the items describing referral report quality

Source of consultation	Consultant N = 40	Specialist N = 78	Resident N = 82	Using Chi Square
**Item**	**No (%)**	**No (%)**	**No (%)**	**P value**
Clinical Findings	26 (65)	55 (70.5)	61 (75.3)	0.55
Laboratory Findings	19 (47.5)	33 (42.3)	57 (70.4)	0.001**
Diagnosis	29 (72.5)	67 (85.9)	67 (81.7)	0.01**
Concise referral aims	35 (87.5)	50 (64.1)	54 (66.7)	0.02**
Degree of urgency	10 (25)	9 (11.5)	19 (23.5)	0.09
Bed side/ otherwise	5 (12.5)	3 (3.8)	7 (8.6)	0.21

Table 3 described the statistical analysis of the items describing referral report quality. It included clinical findings, laboratory findings, mentioning of the diagnosis, written concise aim of referral, degree of urgency of consultation and place of consultation whether at bed side or at consulting physician′s clinic. Good percentage of all categories mentioned clinical findings (P>0.05). All the groups showed very low percentage regarding urgency of consultation and place of consultation (P>0.05). Resident form showed the highest number of laboratory findings (P < 0.01). Specialist form showed the highest number of mentioning the diagnosis (P < 0.01). Consultant form showed the highest number of mentioning the concise aim of referral (P < 0.01).

**Table 3:** analysis of the items describing consultation report quality

Source of consultation	Consultant N = 40	Specialist N = 78	Resident N = 82	Using Chi Square
**Item**	**No (%)**	**No (%)**	**No (%)**	**P value**
**Ethical to**				
Colleagues	19 ( 47.5)	68 (87.2)	51 (63)	0.001**
Patients	31 (77.5)	72 (92.3)	75 (92.6)	0.03*
Patient safety	9 (22.5)	66 (84.6)	38 (47)	0. 001**
Scientific	29 (72.5)	72 (92.3)	61 (75.3)	0. 001**
Differential diagnosis	28 (70)	69 (88.5)	47 (58)	0. 001**
Management plan	36 (90)	73 (93.5)	54 (66.7)	0. 001**

## Discussion

To our knowledge, this study is the first published article about consultation letter quality in Saudi Arabia and even in the Middle East. It shows that the specialists′ consultation letters had the highest percentage of fulfillment of all the six items in the consultation report. There is no uniform existing consultation report (CR) form. The consultation report is semi structured and contains one section for patient pertinent data and a space for consultation. This gave a space for self-composition of the requests by different consultants and allows non uniform reporting. Many CRs failed to ask specific questions, and some were issued for irrelevant medical or surgical conditions.

Consultation reports are an important deliverable in the medical world as important as they are in the business world. Patient care hinges in part on adequate and timely information exchange between treating doctors. Referral and reply letters are common means by which doctors exchange information pertinent to patient care. Ensuring that letters meet the needs of letter recipients saves time for clinicians and patients, reduces unnecessary repetition of diagnostic investigations, and helps to avoid patient dissatisfaction and loss of confidence in medical practitioners.

Aslanger et al. mentioned that the fear of missing important issues leads surgeons to use a decreased threshold for pre-operative consultation requests. Such a non-specific manner of pre-operative consultation request causes unnecessary investigations and decreased cost-effectiveness [7].

Pringle described the referral letter as “the most underexploited method to influence consultant attitudes” and the reply letter “the most neglected route of GP education” [8]. As well as conveying information from one doctor to another, letters also form a valuable source of reference, evidence of the process of informed consent, and a medico-legal record. Some items may have important safety implications. Letters can also help to inform patients, and it will soon be normal practice in the NHS to send copies of letters to patients [9].

In this study, good percentage of all categories mentioned clinical findings (P>0.05). All the groups showed very low percentage regarding urgency of consultation and place of consultation (P>0.05). Resident form showed the highest number of laboratory findings (P < 0.01). Specialist form showed the highest number of mentioning the diagnosis (P < 0.01). Consultant form showed the highest number of mentioning the concise aim of referral (P

The CR need some prior information regarding other medical conditions, such as recent acute attacks of major organ condition, fever, bleeding or medications including ICU admission. It should include sub sectioned areas headed under patient′s history, patient′s physical examination, and results of pertinent laboratory tests, impression to express a professional opinion of the patient′s condition, plan “or” recommendations and conclude the consultation report with a sentence that thanks the referring physician.

In the past decade the desirable content of letters written by consultants have changed little, but the desirable content of general practitioners′ letters has changed somewhat. Despite the views they had expressed, general practitioners frequently did not include “important” items in their referral letters. Nearly all general practitioners considered documentation of medical history and findings both on examination and investigation as important, but these items were documented in only 27-68% of their letters. Consultants′ letters more often contained the items they viewed as desirable, but only about half included what the patient had been told [10].

The patient's medical condition may warrant modification in treatment decisions and should be clarified. Not all patients bring with them complete medical information, and not all self-reported medical histories are reliable [11]. Some of the cases histories can be well explored only with relevant consultants related to some sub specialties. It is preferable for some surgeons to obtain diagnoses from related physicians and then use their own judgment about the need for any management on the basis of current evidence [12].

The consultation letter reflects the diagnostic skills, communication skills, professionalism, and charting management of a physician. It requires synthesis of clinical data, but also reflects distribution of responsibility between providers, professional courtesy, legal requirements, and the writer′s ability to educate regarding a specific case [13, 14]. In this study, there were significant differences between different levels of physicians in fulfillment of consultation letter items. These differences may be due to the trend of consultants towards brevity in writing as they consider that consultant to consultant verbal communication channels are complementary to the written report. Also, specialists; being nearer to the studying and examination and certification, are more reflecting the educational attitude with more item fulfillment. Letters may serve both as correspondence with the referring practitioner, but also are part of the main record keeping tool for the patients.

Some studies have demonstrated improvement in consultation letters secondary to feedback. Fox et al evaluated 15 letters from 5 pediatric consultants, each rated by 1 GP, and 1 pediatric registrar. Three months later, all but one participant showed improvement in overall score [15]. Tattersall, compared letters from 31 oncologists before and after attending a training program which included feedback on their own letters, specific recommendations for content and style of letters, and a prompt card to help with further dictation. There were significant improvements in use of problem lists, headings, and inclusion of specific content items [16].

Conducting a letter-writing training program is an expensive intervention. There are clear advantages of having a structured format for referral and reply letters, including the use of headings to allow the reader to easily identify the information desired [17]. Keely E et al concluded that consultation letter writing being an essential skill for practicing specialists, needs a lot of training. The lack of feedback and education during training, make it a good target for continuing professional development. Peer feedback and self-reflection resulted in long-lasting changes in some individuals [18]. CR training sessions for junior physicians with attendance of senior physicians may be very helpful to present report guidelines, it would reduce the number of unwarranted CRs written by our physicians and it is considered as a mandatory part of the professional development of all the medical career concerned members.

Multiple studies show an insufficient quality in the written communication about patients′ medical situation and in the transferal of duties and obligations from one responsible person or medical team to another [19, 20].

With advancing medical knowledge, there should be continued efforts to update knowledge and skills in communication with consulting professionals in obtaining important medical information so that clinical decisions in the best interest of patients can be made efficiently. For the guidance of healthcare practitioners and the wellbeing of patients, a more rational and consistent approach to defining the desirable content of letters is required. Saudi Med roles of clinicians and health professionals, made this area of communication and consultation an important one within the overall roles needed to be mastered.

## Conclusion

Specialist form showed the highest number of mentioning the diagnosis. Consultant form showed the highest number of mentioning the concise aim of referral. The highest percentage of all categories mentioned all items in consultation report with a good level were the specialists. The introduction of structured medical consultation letter forms led to improvements in obtaining the appropriate information provided by physicians. Training on this important component is highly recommended. Training of the health care providers is also recommended. Satisfaction of the referring physicians from the received consultation letters need also to be assessed.
